# *IL2RG*‐deficient minipigs generated via CRISPR/Cas9 technology support the growth of human melanoma‐derived tumours

**DOI:** 10.1111/cpr.12863

**Published:** 2020-09-01

**Authors:** Jilong Ren, Dawei Yu, Rui Fu, Peipei An, Renren Sun, Zhengzhu Wang, Runfa Guo, Haoyun Li, Ying Zhang, Ziyi Li, Yong‐Guang Yang, Wei Li, Tang Hai, Zheng Hu

**Affiliations:** ^1^ State Key Laboratory of Stem Cell and Reproductive Biology Institute of Zoology Chinese Academy of Sciences Beijing China; ^2^ Institute for Stem Cell and Regeneration Chinese Academy of Sciences Beijing China; ^3^ University of Chinese Academy of Sciences Beijing China; ^4^ Key Laboratory of Organ Regeneration and Transplantation of the Ministry of Education The First Hospital Jilin University Changchun China

**Keywords:** anti‐cancer drug trials, *IL2RG* knockout minipig, in vivo tumour model, tumours, xenogeneic immune rejection

## Abstract

**Objectives:**

Immunodeficient mice injected with human cancer cell lines have been used for human oncology studies and anti‐cancer drug trials for several decades. However, rodents are not ideal species for modelling human cancer because rodents are physiologically dissimilar to humans. Therefore, anti‐tumour drugs tested effective in rodents have a failure rate of 90% or higher in phase III clinical trials. Pigs are similar to humans in size, anatomy, physiology and drug metabolism rate, rendering them a desirable pre‐clinical animal model for assessing anti‐cancer drugs. However, xenogeneic immune rejection is a major barrier to the use of pigs as hosts for human tumours. Interleukin (IL)‐2 receptor γ (IL2RG), a common signalling subunit for multiple immune cytokines including IL‐2, IL‐4, IL‐7, IL‐9, IL‐15 and IL‐21, is required for proper lymphoid development.

**Materials and Methods:**

*IL2RG* ^−/Y^ pigs were generated by CRISPR/Cas9 technology, and examined for immunodeficiency and ability to support human oncogenesis.

**Results:**

Compared to age‐matched wild‐type pigs, *IL2RG* ^−/Y^ pigs exhibited a severely impaired immune system as shown by lymphopenia, lymphoid organ atrophy, poor immunoglobulin function, and T‐ and NK‐cell deficiency. Human melanoma Mel888 cells generated tumours in *IL2RG* ^−/Y^ pigs but not in wild‐type littermates. The human tumours grew faster in *IL2RG* ^−/Y^ pigs than in nude mice.

**Conclusions:**

Our results indicate that these pigs are promising hosts for modelling human cancer in vivo, which may aid in the discovery and development of anti‐cancer drugs.

## 1 INTRODUCTION

Rodent in vivo models, used for several decades in cancer studies, have provided critical insights into the mechanisms of oncogenesis and aided drug and anti‐cancer therapy development.^1^ As a pre‐clinical testing platform, however, rodent cancer models poorly predict responses to anti‐cancer drugs in humans; over 90% of anti‐tumour drugs that are effective in rodents fail in phase III clinical trials.^2^ The extensive evolutionary discrepancy between rodents and humans is likely the main reason that rodents are not well suited to pre‐clinical assessment of human anti‐cancer therapies.^3^, ^4^ This highlights the urgent need for new animal models that can simulate the physiology and pathology involved in human cancer, thereby improving the efficiency of the pre‐clinical development of anti‐cancer agents and therapies.^5^

Unlike rodents, swine are similar to humans in body size, anatomy and physiology.^6^ Pigs were once even considered suitable as xeno‐organ donors for clinical organ transplantation into humans.^7^, ^8^, ^9^ The pig lifespan is much longer than that of rodents and more similar to that of humans. This is advantageous for studying relapse and qualifying the long‐term efficacy of anti‐cancer therapeutics.^10^, ^11^ Pigs and humans also show similar profiles of drug metabolism, which is important for accurately evaluating the effects of anti‐cancer drugs.^12^ Several models of spontaneously occurring cancer have been established in pigs recently via modification of the pig genetic background.^13^, ^14^, ^15^ Although cancer in pigs differs from that in humans, developing pig strains that can host human cancer cells will help establish pre‐clinical models for the assessment of anti‐cancer regimens.

Successful transfer of human cancer cells or tumour tissues into pigs is challenging because of their robust T‐cell‐mediated xenogeneic rejection response.^16^, ^17^ Interleukin (IL)‐2 receptor γ (IL2RG) is a common cytokine subunit shared by a number of interleukins—including IL‐2, IL‐4, IL‐7, IL‐9, IL‐15 and IL‐21—that are critical for the development and survival of immune cell subsets such as T and NK cells.^18^, ^19^ Previous studies have shown that *IL2RG* ^−/Y^ pigs, which have functionally defective T and NK cells, tolerate allogeneic pig bone marrow transplantation without the use of immunosuppressive agents.^20^ However, the ability of *IL2RG* ^−/Y^ pigs to host human cancer cells remains unclear.

In this study, we used CRISPR/Cas9 technology to generate Bama *IL2RG* ^−/Y^ pigs to assess whether they are suitable as an in vivo model for the study of human cancers. This work will facilitate the development of pig strains that can host human cancer cells to establish pre‐clinical models for the assessment of anti‐cancer regimens.

## 2 MATERIALS AND METHODS

### 2.1 Animal care and use

Bama miniature pigs were obtained from and housed at Beijing Farm Animal Research Center of the Chinese Academy of Science. Nude mice were obtained from Vital River Laboratories and housed in the Laboratory Animal Center of the Chinese Academy of Sciences. All procedures involving animals were carried out in accordance with the Guidelines for the Use of Animals in Research, issued by the Institute of Zoology of the Chinese Academy of Sciences.

### 2.2 Derivation of an *IL2RG* ^−/Y^ fibroblast cell line

Porcine foetal fibroblasts (PFF) were isolated from a Bama miniature foetus at day 35 of gestation and cultured in high‐glucose DMEM (Gibco) supplemented with 15% foetal bovine serum (Gibco), 1 × non‐essential amino acids (Gibco), 2 mmol/L L‐glutamine (Gibco), 100 units/mL penicillin (Life Technologies) and 100 μg/mL streptomycin (Life Technologies) at 38.5°C in 5% CO_2_. We co‐transfected Cas9‐GFP and gRNA vectors into the cultured PFF (P3) via nucleofection (Lonza, Germany). At 36‐48 hours post‐transfection, the cells were harvested using 0.05% trypsin (Gibco). A single GFP‐positive cell was plated in each well of a 96‐well plate via fluorescence‐activated cell sorting and cultured for approximately 7‐12 days in previous cell culture medium. Colonies grown from each single cell were used for subsequent genetic analysis and nuclear transfer.

### 2.3 Nuclear and embryo transfer

We performed nuclear transfer as described previously.^21^ In brief, we enucleated oocytes in metaphase II via aspiration of the first polar body and the adjacent cytoplasm using a bevelled glass pipette in MAN medium containing 7.5 μg/mL cytochalasin B. A morphologically qualified donor cell was injected into the perivitelline space and placed adjacent to the recipient cytoplasm. We induced fusion and activation of the karyoplast‐cytoplast complexes in fusion medium (0.3 mol/L mannitol, 1.0 mmol/L CaCl_2_, 0.1 mmol/L MgCl_2_ and 0.5 mmol/L Hepes [pH adjusted to 7.0‐7.4]) with two 30‐microsecond pulses (administered at 1‐second intervals) of direct current at 1.2 kV/cm generated via Eppendorf Multiporator. The oocytes were then incubated for 20 minutes in porcine zygote medium 3 (PZM‐3) and evaluated for fusion under a stereomicroscope. We placed the fused embryos into 4‐well cell culture plates containing 500 μL PZM‐3/well and incubated at 38.5°C in an atmosphere comprising 5% CO_2_, 5% O_2_ and 90% air. The day after nuclear transfer, we transferred 2‐cell somatic cell nuclear transfer embryos into the oviducts of surrogates on the day of, or 1 day after, the onset of oestrus. Pregnancy was determined at approximately day 30, and surrogates were assessed regularly at 1‐month intervals via ultrasound examination. All cloned piglets were delivered by natural birth on day 114 of gestation.

### 2.4 Xenografting of tumour cells

We cultured human Mel888 melanoma cells in DMEM supplemented with 10% foetal bovine serum, 1 × non‐essential amino acids, 2 mmol/L L‐glutamine, 100 units/mL penicillin and 100 μg/mL streptomycin (all from Gibco) at 37°C in a humidified atmosphere containing 5% CO_2_. After the cells reached 80%‐100% confluence, they were trypsinized (Gibco), washed, counted and resuspended at a concentration of 1 × 10^7^ cells/mL in a 1:1 mixture of PBS and Matrigel (Corning Life Sciences). The pigs (5 wild‐type and 3 *IL2RG* ^−/Y^ pigs) were anesthetized with isoflurane and injected subcutaneously on the right abdomen near the groin with 1 mL (1 × 10^7^ Mel888 cells) of cell suspension. We injected nude mice with the same cell suspension, volume and procedure as those used for the pigs. We evaluated the tumours in the pigs and mice every 5 days.

### 2.5 Enzyme‐linked immunosorbent assay

The serum levels of porcine immunoglobulin were measured using anti‐pig IgM, anti‐pig IgG and anti‐pig IgA enzyme‐linked immunosorbent assay (ELISA) kits in accordance with the manufacturer's instructions (Bethyl Laboratories).

### 2.6 Flow cytometry

Peripheral blood mononuclear cells and splenocytes were harvested from wild‐type and *IL2RG* ^−/Y^ pigs, and immunolabelled with anti‐pig CD3, anti‐pig CD4, anti‐pig CD8, anti‐pig CD45RA, anti‐pig CD16 and anti‐pig CD14 primary antibodies (BD, Franklin Lakes, NJ, USA) in accordance with the manufacturer's protocols. The samples were processed by flow cytometry, and the data were analysed using FlowJo software (BD).

### 2.7 Haematoxylin and eosin staining

Porcine lymphoid organs, including the thymus, spleen and lymph nodes, were collected from wild‐type and *IL2RG* ^−/Y^ pigs, fixed in 10% buffered formalin (Sangon Biotech), embedded in paraffin, sectioned into 4‐μm slices (Leica, Wetzlar, Germany) and stained with haematoxylin and eosin (H&E; Thermo Fisher).

### 2.8 Statistical analysis

Statistical analyses were performed using GraphPad Prism software (GraphPad Prism 7.0). Each experiment included at least 3 independent biological replicates. For statistical comparisons, we used Student's *t* test and one‐way analysis of variance. All data are presented as the mean ± standard error of the mean. Statistically significant differences are indicated in the figures and text as follow: *P < .05; **P < .01; and ***P < .001. The statistical parameters for specific experiments, including the type of analysis, statistical significance and n values, are reported in the figure legends.

## 3 RESULTS

### 3.1 Establishment of the *IL2RG* ^−/Y^ pig model

**FIGURE 1:**
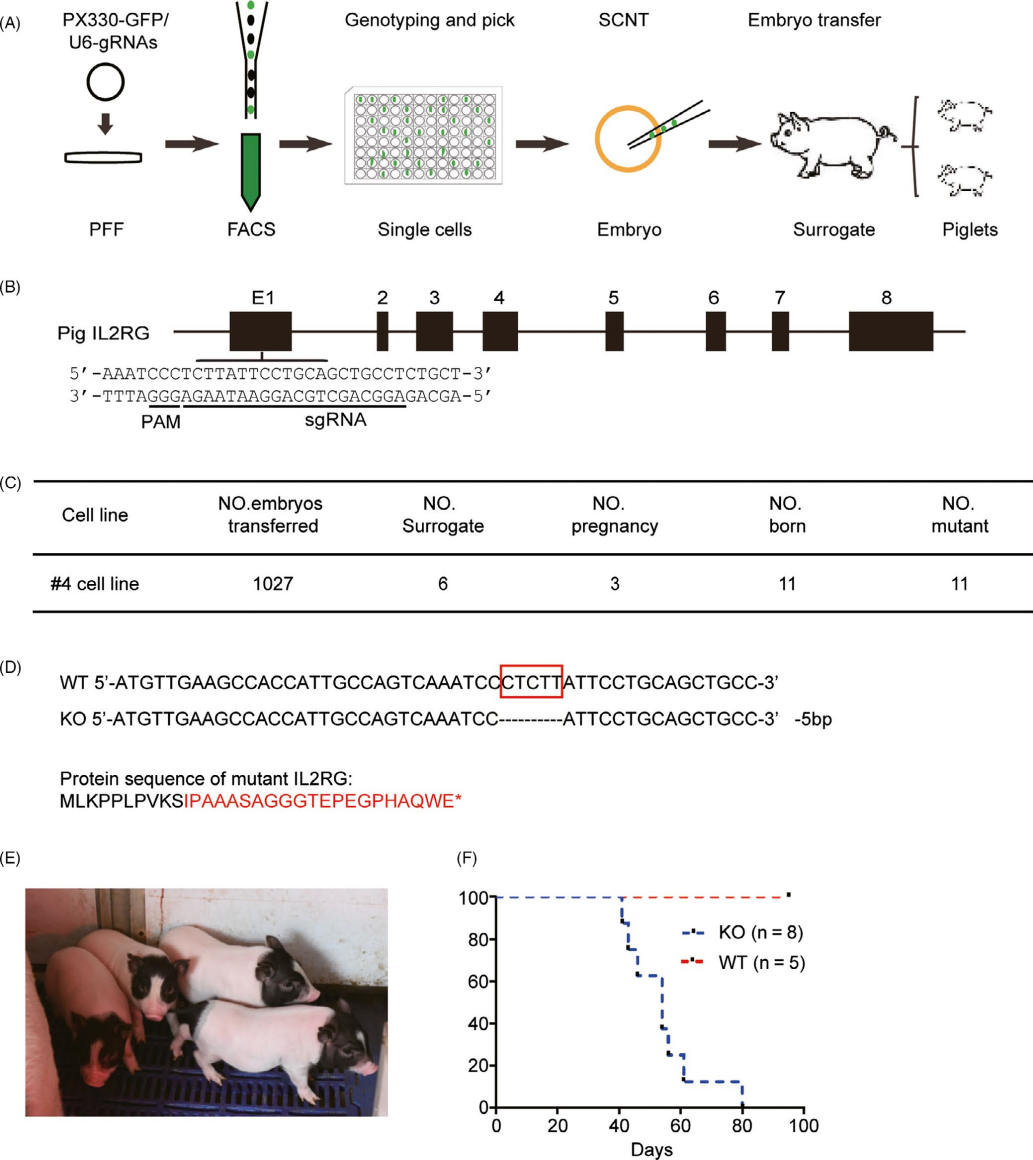
Use of the CRISPR/Cas9 system to generate *IL2RG*‐deficient pigs. Schematic illustrating the generation of *IL2RG*‐deficient pigs. The *IL2RG* knockout cell lines were established from single cells derived by CRISPR/Cas9 nucleofection of PFF. A, Somatic cell nuclear transfer was performed to generate *IL2RG* knockout pigs. B, Design of specific CRISPRs used to target *IL2RG*. Underlined sequences indicate target sequences and protospacer adjacent motif sequences. C, Genotype of *IL2RG* knockout pigs. Red box indicates the deleted nucleotides. Predicted sequence of amino acids after knockout; red font highlights differences from the wild‐type sequence and * indicates termination. D, *IL2RG* knockout cell line 4 was chosen for nuclear transfer. A total of 1,027 cloned embryos were reconstructed from this cell line, and pregnancy was confirmed in 3 of the 6 surrogates. Eleven piglets were born to the surrogates. E, The piglets were raised in a normal environment. F, Survival curve for the piglets. The data are presented as the mean ± standard error of the mean. Statistical significance, determined by Student's *t* test, is indicated by *, *P* < .05; **, *P* < .01; and ***, *P* < .001

A schematic representation of the method used to generate *IL2RG* knockout pigs is shown in Figure 1A. The pig *IL2RG* gene is located on the X chromosome and contains 8 exons. A suitable protospacer adjacent motif target sequence was found in the first exon; the single‐guide RNA sequence is shown in Figure 1B. We co‐transfected the Cas9 and all sgRNA vectors into pig PFF, and plated Cas9‐positive cells in each well of 96‐well plates by fluorescence‐activated cell sorting. After polymerase chain reaction genotyping and sequencing, we selected *IL2RG* knockout cell line 4 for nuclear transfer. We reconstructed 1,027 cloned embryos from this cell line, with which 6 recipients were transplanted at 35 to 40 days after embryo transfer. We detected pregnancy in 3 of the 6 surrogates by B‐mode ultrasound. Eleven piglets were born to these 3 surrogates (Figure 1C). We extracted DNA from the ear tissue of the piglets to confirm the knockout of *IL2RG*. We found that all 11 piglets had a 5‐bp deletion in *IL2RG* exon 1; the predicted protein sequence of mutant IL2RG is shown in Figure 1D. To foster resistance to infection in an open environment, the piglets were nursed by their mothers and surrogate milk pigs for as long as possible. Three piglets from the first litter died immediately after birth due to hypoxia; the rest of the piglets were healthy (Figure 1E). The lifespan of the piglets was approximately 40‐60 days (Figure 1F).

### 3.2 Immune system of the IL2RG^−/Y^ pig

**FIGURE 2:**
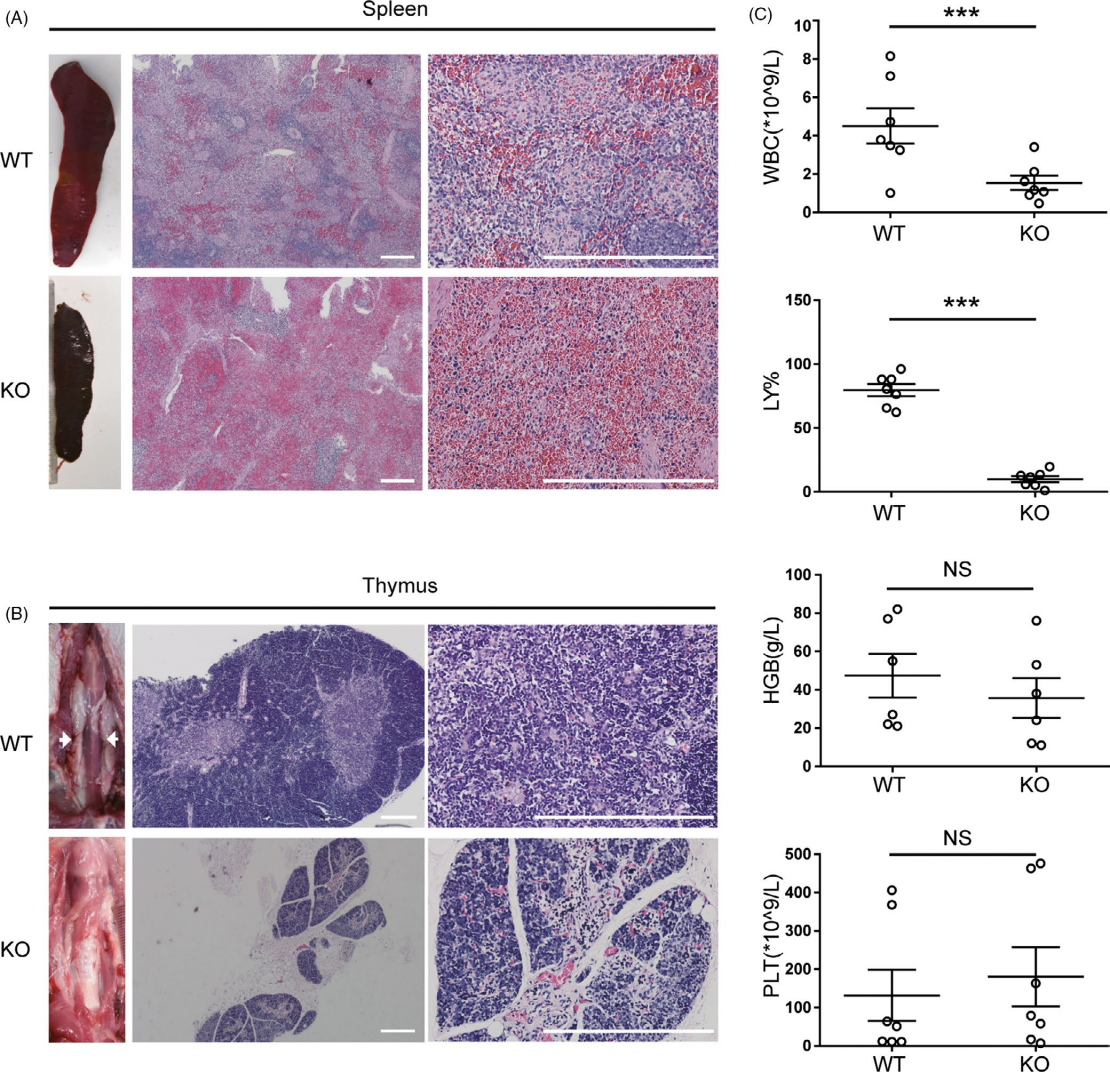
Severe combined immunodeficiency phenotype of *IL2RG*‐deficient pigs. A, The thymi of *IL2RG* knockout pigs were atrophied compared with the normal thymi of wild‐type pigs. Representative images of H&E‐stained thymus tissue, showing lack of lymphocytes within the thymus of an *IL2RG*‐deficient pig and lymphocytes in the thymus of wild‐type pigs. B, *IL2RG* knockout pigs are athymic whereas normal thymi are present in wild‐type pigs. C, Comprehensive metabolic analysis of 10‐day‐old *IL2RG* knockout and wild‐type control piglets. Scale bar, 200 µm. The data are presented as the mean ± standard error of the mean. Statistical significance, determined by Student's *t* test, is indicated by *P < .05; **P < .01; and ***P < .001. NS: not significant

To assess the phenotype of the IL2RG^−/Y^ pig, we analysed the morphology of the spleen, thymus, liver, lung and lymph nodes via H&E staining. Compared with wild‐type piglets, the *IL2RG* ^−/Y^ piglets had clear dysplasia of the spleen with few, or missing, lymph nodes. The lymphatic sheath around the central artery of the spleen was dysplastic, the content of the white pulp was reduced, and the number of lymphocytes in the spleen was significantly lower in *IL2RG* ^−/Y^ piglets compared to wild‐type animals (Figure 2A; Figure S2). Most of the *IL2RG* ^−/Y^ piglets were athymic or possessed severely atrophied thymi. Compared with that of wild‐type piglets, the thymus structure of the *IL2RG* ^−/Y^ piglets was abnormal, with poorly developed thymic corpuscles (Figure 2B). At 10 days of age, *IL2RG* ^−/Y^ pigs had significantly lower numbers of white blood cells in their peripheral blood than 10‐day‐old wild‐type pigs. Although the proportion of lymphocytes among the blood cells was significantly lower in *IL2RG* ^−/Y^ pigs, the red blood cell and platelet counts did not statistically differ in the *IL2RG* ^−/Y^ pigs and wild‐type controls (Figure 2C). The biochemical parameters of the livers and lungs of the immunodeficient pigs did not statistically differ from those of wild‐type controls (Figure S2).

**FIGURE 3:**
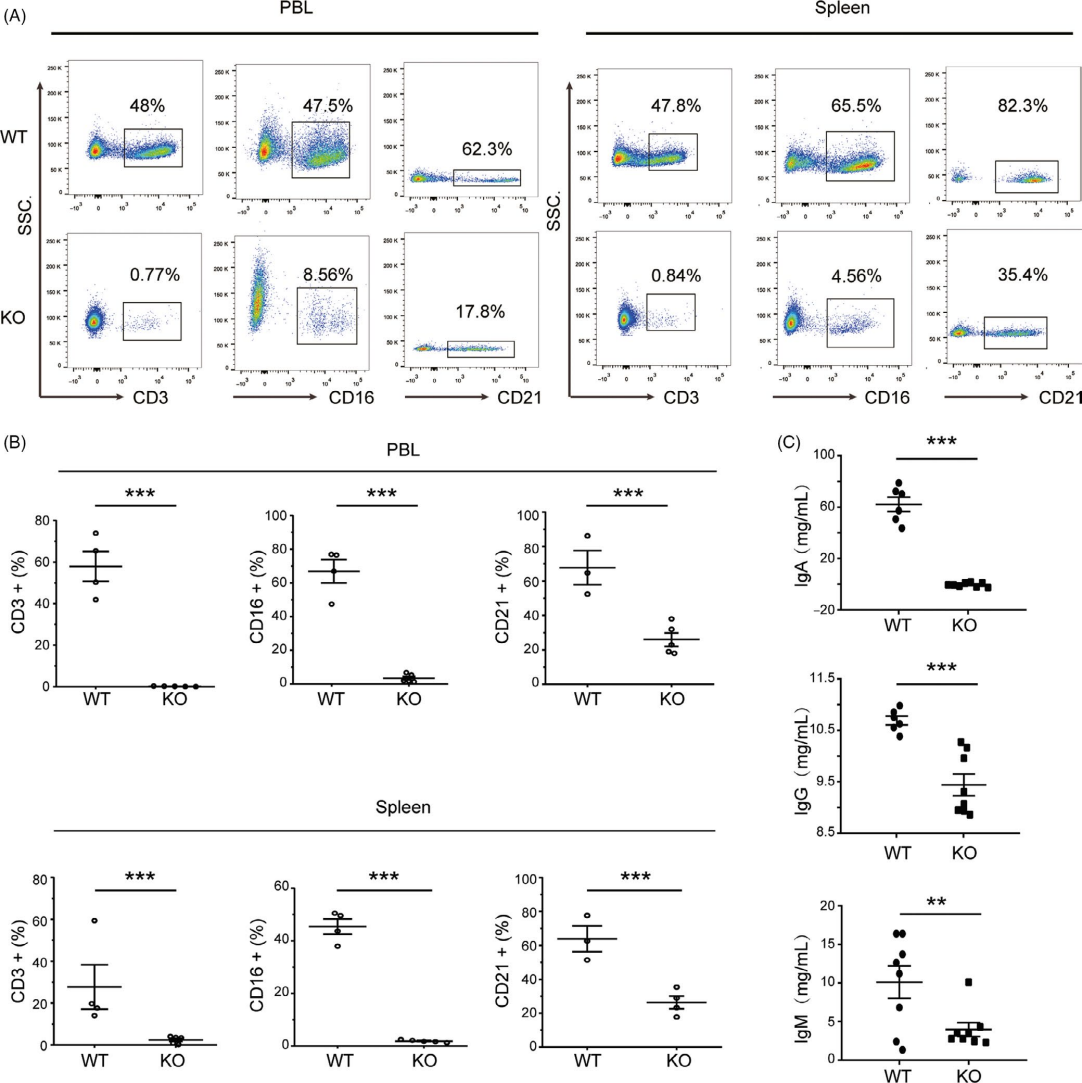
Lymphocyte and immunoglobulin analyses by flow cytometry and ELISA. A, Flow cytometric analysis of T, B and NK cells in the peripheral blood and spleens of *IL2RG* knockout and wild‐type pigs. Dot plots show the CD3, CD21 and CD16 immunolabelling used to differentiate subpopulations of T, B and NK cells, respectively, in peripheral blood. B, Proportions of T, B and NK cells among the peripheral blood mononuclear cells and splenocytes of wild‐type (n = 4) and *IL2RG*‐deficient pigs (n = 5). C, Immunoglobulin titres were determined by ELISA in wild‐type (n = 8) and *IL2RG*‐deficient (n = 8) pig sera. The data are presented as the mean ± standard error of the mean. Statistical significance, determined by Student's *t* test, is indicated by *P < .05; **P < .01; and ***P < .001. NS: not significant

We immunolabelled lymphocytes with anti‐pig CD45 antibody to analyse the immune cell populations of the knockout animals. Flow cytometric analysis of peripheral blood showed that the percentage of CD3‐positive T cells among the CD45‐positive cells in *IL2RG* ^−/Y^ pigs (0.14 ± 0.04%) was significantly lower than that in wild‐type pigs (57.87 ± 7.22%; *P* < .0001). The percentage of CD3‐negative and CD16‐positive NK cells in *IL2RG* ^−/Y^ pigs (3.41 ± 1.10%) was also significantly lower than that in wild‐type pigs (66.93 ± 6.18%; *P* < .0001). The CD3‐negative and CD21‐positive B‐cell population was also less abundant in the *IL2RG*‐deficient pigs (26.00 ± 3.89%) than in wild‐type pigs (67.77 ± 7.65%; *P* = .003). We next investigated the splenic lymphocyte composition of the pigs using flow cytometry. The percentage of T cells in the spleens of *IL2RG* ^−/Y^ pigs was 2.41 ± 0.78%, whereas that in wild‐type piglets was 27.69 ± 9.51% (*P* < .0001). The percentage of B cells in the spleens of *IL2RG* ^−/Y^ pigs was 26.38 ± 3.38%, whereas that in wild‐type controls was 63.87 ± 5.90% (*P* = .004). Finally, the percentage of NK cells in the spleens of *IL2RG* ^−/Y^ pigs was 1.84 ± 0.21% and the percentage in wild‐type controls was 45.45 ± 2.60% (*P* < .0001). Thus, *IL2RG* ^−/Y^ pigs have lower percentages of T, NK and B cells in their peripheral blood and spleens than wild‐type controls (Figure 3A, B).

We used ELISA to assess immunoglobulin levels in the peripheral blood of *IL2RG* ^−/Y^ pigs and wild‐type controls. We found that *IL2RG* ^−/Y^ pigs had lower levels of IgM, IgA and IgG in their peripheral blood than wild‐type controls (IgM: 3.96 ± 0.91 versus 12.61 ± 1.47 mg/mL, respectively [*P* = .0001]; IgA: 0.44 ± 0.55 versus 62.08 ± 5.6 mg/mL, respectively [*P* < .0001]; and IgG: 9.44 ± 0.21 versus 10.76 ± 0.09 mg/mL, respectively [*P* = .0003]; Figure 3C). These results indicate that the *IL2RG* ^−/Y^ pigs were severely immune deficient.

### 3.3 Human tumour growth in *IL2RG* ^−/Y^ pigs

**FIGURE 4:**
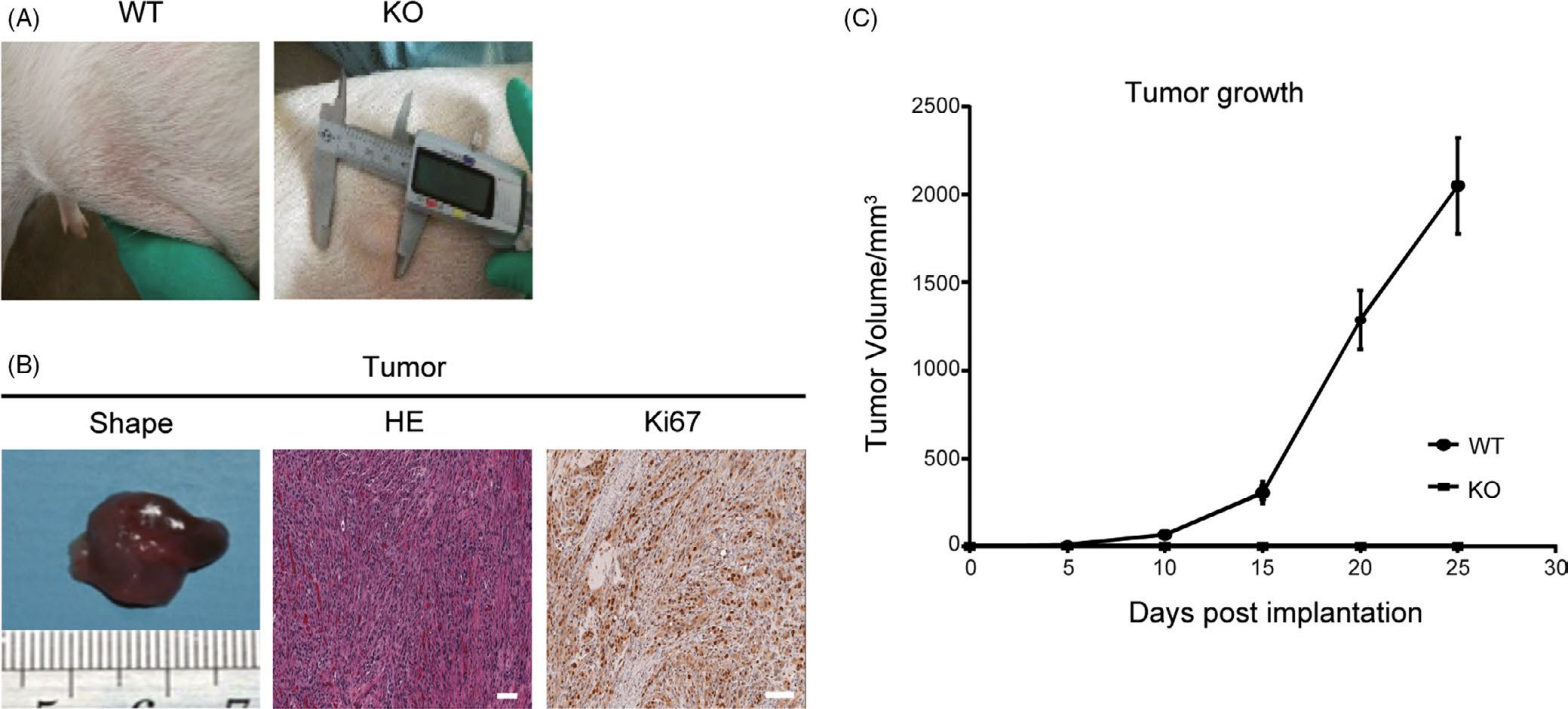
Tumour formation after injection of Mel888 cells into the abdomens of *IL2RG*‐deficient pigs. A, Tumour formation was observed in the abdomens of *IL2RG* knockout pigs 20 days after the injection of Mel888 tumour cells. B, H&E staining indicated that the tumours showed typical tumour structure. More than 20% of tumour cells were Ki67‐positive. C, Curves showing tumour growth within 25 days of subcutaneous injection of tumour cells into *IL2RG*‐deficient and wild‐type pigs. Scale bar, 200 µm. The data are presented as the mean ± standard error of the mean. Statistical significance was determined by Student's *t* test

To evaluate whether *IL2RG* ^−/Y^ pigs can be used as an in vivo model for human oncology studies, we inoculated Mel888 cells into *IL2RG* ^−/Y^ pigs and immunocompetent wild‐type controls. We subcutaneously injected 1x10^7^ cells into the abdomens of 25‐day‐old piglets and measured tumour size every 5 days. *IL2RG* ^−/Y^ pigs bearing human tumours were sacrificed at day 25 after injection, and the engrafted human tumours were harvested and examined by H&E staining. We found that none of the immunocompetent control pigs successfully supported human tumour growth, whereas we observed formation of human tumours in 3 of the 4 *IL2RG* ^−/Y^ pigs as early as 10 days post‐injection, followed by continued tumour growth (Figure 4A). The H&E results showed that the engrafted human melanoma tumours showed scant infiltration by porcine lymphocytes, indicating that *IL2RG* ^−/Y^ pigs were unable to reject the solid human tumours. Over 20% of the tumour cells were Ki67‐positive after immunolabelling, indicating robust tumour growth (Figure 4B). To compare the utility of this pig model with that of the nude mouse model, we injected *IL2RG* ^−/Y^ pigs and nude mice with the same number of Mel888 cells. We found that the tumour growth rate and size in the *IL2RG* ^−/Y^ pig model were superior to those observed in the nude mouse model (Figure 4C; Figure S1A‐C). These findings indicate that *IL2RG* ^−/Y^ pigs can be used as an in vivo model for human oncology studies.

## 4 DISCUSSION

Animal models that support the growth of human tumours have facilitated human oncology studies and anti‐cancer drug discovery in the past few decades. Although immunodeficient mice are commonly used for the study of human cancer, physiologic discrepancies between rodents and humans necessitate the development of more suitable in vivo animal models for human oncology investigations. In this study, we used CRISPR/Cas9 technology to develop an immunocompromised pig model via knockout of *IL2RG*. The *IL2RG* ^−/Y^ pigs within 60 days after birth had atrophied primary and secondary lymphoid organs with no typical lymphoid structures, such as the cortex in the thymus or white pulp in the spleen. The haemoglobulin levels and platelet counts were comparable in the *IL2RG* ^−/Y^ pigs and wild‐type counterparts. However, white blood cell counts, especially lymphocyte counts, were statistically lower in *IL2RG* ^−/Y^ pigs than in wild‐type controls. In agreement with previous reports, mature T cells and NK cells were largely absent from *IL2RG* ^−/Y^ pigs.^20^, ^22^ Although B cells were detected in the *IL2RG* ^−/Y^ pigs, the levels of IgM, IgG and IgA were significantly lower in *IL2RG* ^−/Y^ pigs than in wild‐type controls of similar age. These findings indicate considerable impairment of the humoral system. Most importantly, we found that *IL2RG* ^−/Y^ pigs supported the growth of human cancer cells and the formation of human tumours. We observed only scant infiltration of porcine immune cells into the engrafted human tumours. Considering pig adaptive and innate immune system has already well developed in the wild‐type control Bamma piglets which rapidly rejected implanted human cancer cells, these data indicating that *IL2RG* ^−/Y^ pigs are a suitable in vivo model for human cancer.

Disruptions in IL2RG expression impair the development of the immune system in various species, including rodents,^23^ humans ^19^ and pigs.^20^, ^22^ In agreement with previous reports, our study shows that few mature porcine T or NK cells, which play crucial roles in xenogeneic rejection, were found in *IL2RG* ^−/Y^ pigs. Unsurprisingly, we observed only rare infiltration of porcine lymphocytes into engrafted human tumours due to the compromised immune systems in *IL2RG* ^−/Y^ pigs. Xeno‐reactive antibodies and macrophages can also mediate xenogeneic rejection. Although lymph node atrophy and the absence of porcine T cells markedly inhibit xenogeneic humoral immune responses, porcine B cells can still secrete anti‐human IgM antibodies, which may lyse human cells with the help of the porcine complement system. Macrophages also play critical roles in xenogeneic rejection because of the incompatibility of host SIRPα and donor CD47 molecules. Although macrophage‐mediated rejection alone does not interfere with xenogeneic solid tumour formation, it can interfere with the engraftment of xeno‐haematological malignancies.^24^, ^25^, ^26^ Therefore, the development of novel immunodeficient porcine strains lacking an anti‐human humoral response and macrophage‐mediated rejection will facilitate the study of human oncogenesis.

Various immunodeficient mouse strains support the replication of primary human tumours.^27^, ^28^, ^29^ However, the vast physiologic differences between rodents and humans limit the usefulness of human tumour‐bearing mouse models. Human tumours formed in mice are usually much smaller than those in humans, which interferes with the prediction of the tumour responses to anti‐tumour drugs. The development and relapse of cancer in humans may occur over years or even decades; however, the average lifespan of a mouse is less than 3 years. Therefore, mice are used mostly to model aggressive, rapidly expanding human tumours. Moreover, the physiological differences between human and rodents impede the use of mouse models for human oncology studies. Compared to rodents, pigs share more similarities with humans in terms of anatomy, physiology and metabolism. The development of pig models that can support the growth of human tumours will help to overcome the limitations of the currently available mouse models.

Short lifespans of these immunodeficient piglets, which mainly caused by infections in an open environment, limit their application in long‐term predictive studies for anti‐cancer drugs, such as like Braf/MEK inhibitor, or analysing of additional human cancers in different anatomical locations (eg lungs and colon). Establishment of a standard procedure, such as caesarean delivery, to promote immunodeficient pig long‐term survival in decontaminated facilities is an important problem to be resolved.

## CONFLICT OF INTERESTS

The authors declare that they have no competing interests.

## AUTHOR CONTRIBUTIONS

HT and HZ conceived and supervised the study. RJ, YD, FR,AP, SR, WZ,GR and LH designed and performed most of the experiments. RJ, ZY and HZ analysed and discussed the data. HZ, HT, RJ and FR wrote the manuscript. LW, YY and LZ revised the manuscript. All authors edited the manuscript and approved it for submission.

## ETHICAL APPROVAL

This study was approved by the Ethical Review Committee of the Institute of Zoology at the Chinese Academy of Sciences (Beijing, China).

## Supporting information

Figure S1

Figure S2

## Data Availability

The data that support the findings of this study are available from the corresponding author upon reasonable request.

## References

[1] Mouse Genome Sequencing Consortium , et al. Initial sequencing and comparative analysis of the mouse genome. Nature. 2002;420(6915):520‐562. 12466850 10.1038/nature01262

[2] Oehler MK , Bicknell R . The promise of anti‐angiogenic cancer therapy. Br J Cancer. 2000;82(4):749‐752. 10732738 10.1054/bjoc.1999.0991 PMC2374416

[3] McGonigle P , Ruggeri B . Animal models of human disease: challenges in enabling translation. Biochem Pharmacol. 2014;87(1):162‐171. 23954708 10.1016/j.bcp.2013.08.006

[4] Mestas J , Hughes CC . Of mice and not men: differences between mouse and human immunology. J Immunol. 2004;172(5):2731‐2738. 14978070 10.4049/jimmunol.172.5.2731

[5] Li XJ , Li W . Beyond mice: genetically modifying larger animals to model human diseases. J Genet Genomics. 2012;39(6):237‐238. 22749009 10.1016/j.jgg.2012.05.006

[6] Walters EM , Agca Y , Ganjam V , Evans T . Animal models got you puzzled?: think pig. Ann N Y Acad Sci. 2011;1245:63‐64. 22211982 10.1111/j.1749-6632.2011.06345.x

[7] Iwase H , Liu H , Wijkstrom M , et al. Pig kidney graft survival in a baboon for 136 days: longest life‐supporting organ graft survival to date. Xenotransplantation. 2015;22(4):302‐309. 26130164 10.1111/xen.12174 PMC4519393

[8] Iwase H , Liu H , Schmelzer E , et al. Transplantation of hepatocytes from genetically engineered pigs into baboons. Xenotransplantation. 2017;24(2):e12289. 10.1111/xen.12289 PMC5397320 28130881

[9] Zhang R , Wang Y , Chen L , et al. Reducing immunoreactivity of porcine bioprosthetic heart valves by genetically‐deleting three major glycan antigens, GGTA1/β4GalNT2/CMAH. Acta Biomater. 2018;72:196‐205. 29631050 10.1016/j.actbio.2018.03.055

[10] Flisikowska T , Kind A , Schnieke A . Pigs as models of human cancers. Theriogenology. 2016;86(1):433‐437. 27156684 10.1016/j.theriogenology.2016.04.058

[11] Neff EP . Cancer modeling thinks big with the pig. Lab Anim (NY). 2019;48(3):75‐78. 30742111 10.1038/s41684-019-0246-5

[12] Liu Y , Zeng BH , Shang HT , Cen YY , Wei H . Bama miniature pigs (*Sus scrofa domestica*) as a model for drug evaluation for humans: comparison of in vitro metabolism and in vivo pharmacokinetics of lovastatin. Comp Med. 2008;58(6):580‐587. 19149415 PMC2710758

[13] Overgaard NH , Frøsig TM , Welner S , et al. Establishing the pig as a large animal model for vaccine development against human cancer. Front Genet. 2015;6:286. 26442104 10.3389/fgene.2015.00286 PMC4584933

[14] Luo Y , Li J , Liu Y , et al. High efficiency of BRCA1 knockout using rAAV‐mediated gene targeting: developing a pig model for breast cancer. Transgenic Res. 2011;20(5):975‐988. 21181439 10.1007/s11248-010-9472-8

[15] Kurahashi M , Miyake H , Takagi T , Tashiro S . Changes of lymphatic flow in case of pancreatic duct obstruction in the pig–as a model of pancreatic cancer. J Med Invest. 2004;51(1–2):70‐75. 15000259 10.2152/jmi.51.70

[16] Ulrichs K , Eckstein V , Müller‐Ruchholtz W . Xenogeneic T‐cell‐mediated immune reactivity in the model of pig‐to‐humans: first findings with native stimulator cells. Transplant Proc. 1994;26(2):1045‐1046. 8171462

[17] Geczy AF , de Weck AL . Molecular basis of T cell dependent genetic control of the immune response in the guinea pig. Prog Allergy. 1977;22:147‐213. 141047

[18] Tassara C , Pepper AE , Puck JM . Intronic point mutation in the IL2RG gene causing X‐linked severe combined immunodeficiency. Hum Mol Genet. 1995;4(9):1693‐1695. 8541866 10.1093/hmg/4.9.1693

[19] Puck JM , Pepper AE , Henthorn PS , et al. Mutation analysis of IL2RG in human X‐linked severe combined immunodeficiency. Blood. 1997;89(6):1968‐1977. 9058718

[20] Suzuki S , Iwamoto M , Saito Y , et al. Il2rg gene‐targeted severe combined immunodeficiency pigs. Cell Stem Cell. 2012;10(6):753‐758. 22704516 10.1016/j.stem.2012.04.021

[21] Song Y , Hai T , Wang Y , et al. Epigenetic reprogramming, gene expression and in vitro development of porcine SCNT embryos are significantly improved by a histone deacetylase inhibitor–m‐carboxycinnamic acid bishydroxamide (CBHA). Protein Cell. 2014;5(5):382‐393. 24627095 10.1007/s13238-014-0034-3 PMC3996156

[22] Kang J‐T , Cho B , Ryu J , et al. Biallelic modification of IL2RG leads to severe combined immunodeficiency in pigs. Reprod Biol Endocrinol. 2016;14(1):74. 27809915 10.1186/s12958-016-0206-5 PMC5095964

[23] Azuma H , Paulk N , Ranade A , et al. Robust expansion of human hepatocytes in Fah‐/‐/Rag2‐/‐/Il2rg‐/‐ mice. Nat Biotechnol. 2007;25(8):903‐910. 17664939 10.1038/nbt1326 PMC3404624

[24] Willingham SB , Volkmer J‐P , Gentles AJ , et al. The CD47‐signal regulatory protein alpha (SIRPa) interaction is a therapeutic target for human solid tumors. Proc Natl Acad Sci U S A. 2012;109(17):6662‐6667. 22451913 10.1073/pnas.1121623109 PMC3340046

[25] Horrigan SK , Reproducibility Project: Cancer Biology . Replication Study: The CD47‐signal regulatory protein alpha (SIRPa) interaction is a therapeutic target for human solid tumors. eLife. 2017;6:e18173. 28100392 10.7554/eLife.18173 PMC5245970

[26] Chroscinski D , Maherali N , Griner E , Reproducibility Project: Cancer Biology . Registered report: The CD47‐signal regulated protein alpha (SIRPa) interaction is a therapeutic target for human solid tumors. eLife. 2015;4:e04586. 10.7554/eLife.04586 PMC4383318 25621565

[27] Brehm MA , Cuthbert A , Yang C , et al. Parameters for establishing humanized mouse models to study human immunity: analysis of human hematopoietic stem cell engraftment in three immunodeficient strains of mice bearing the IL2rgamma(null) mutation. Clin Immunol. 2010;135(1):84‐98. 20096637 10.1016/j.clim.2009.12.008 PMC2835837

[28] Carreno BM , Garbow JR , Kolar GR , et al. Immunodeficient mouse strains display marked variability in growth of human melanoma lung metastases. Clin Cancer Res. 2009;15(10):3277‐3286. 19447870 10.1158/1078-0432.CCR-08-2502 PMC2697956

[29] Silva‐Barbosa SD , Butler‐Browne GS , Di Santo JP , Mouly V . Comparative analysis of genetically engineered immunodeficient mouse strains as recipients for human myoblast transplantation. Cell Transplant. 2005;14(7):457‐467. 16285254 10.3727/000000005783982837

