# Supplementary figures and images for: IL2RG‐deficient minipigs generated via CRISPR/Cas9 technology support the growth of human melanoma‐derived tumours
Source: Cell Prolif. 2020 Sep 1;53(10):e12863. doi: 10.1111/cpr.12863 (PMC7574875; doi:10.1111/cpr.12863)

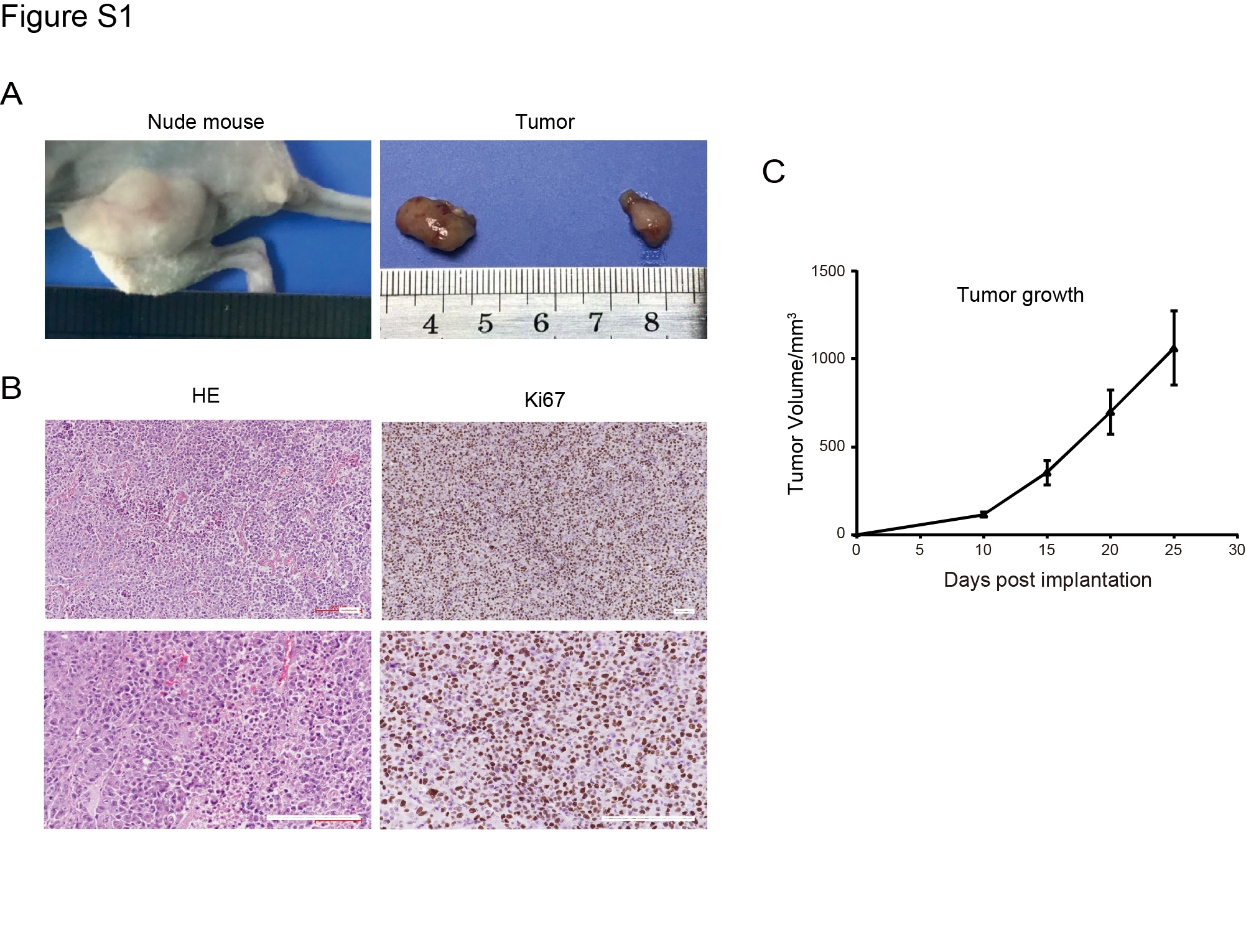

Supplement: Supplementary file 1 — Figure S1 [file CPR-53-e12863-s001.jpg]

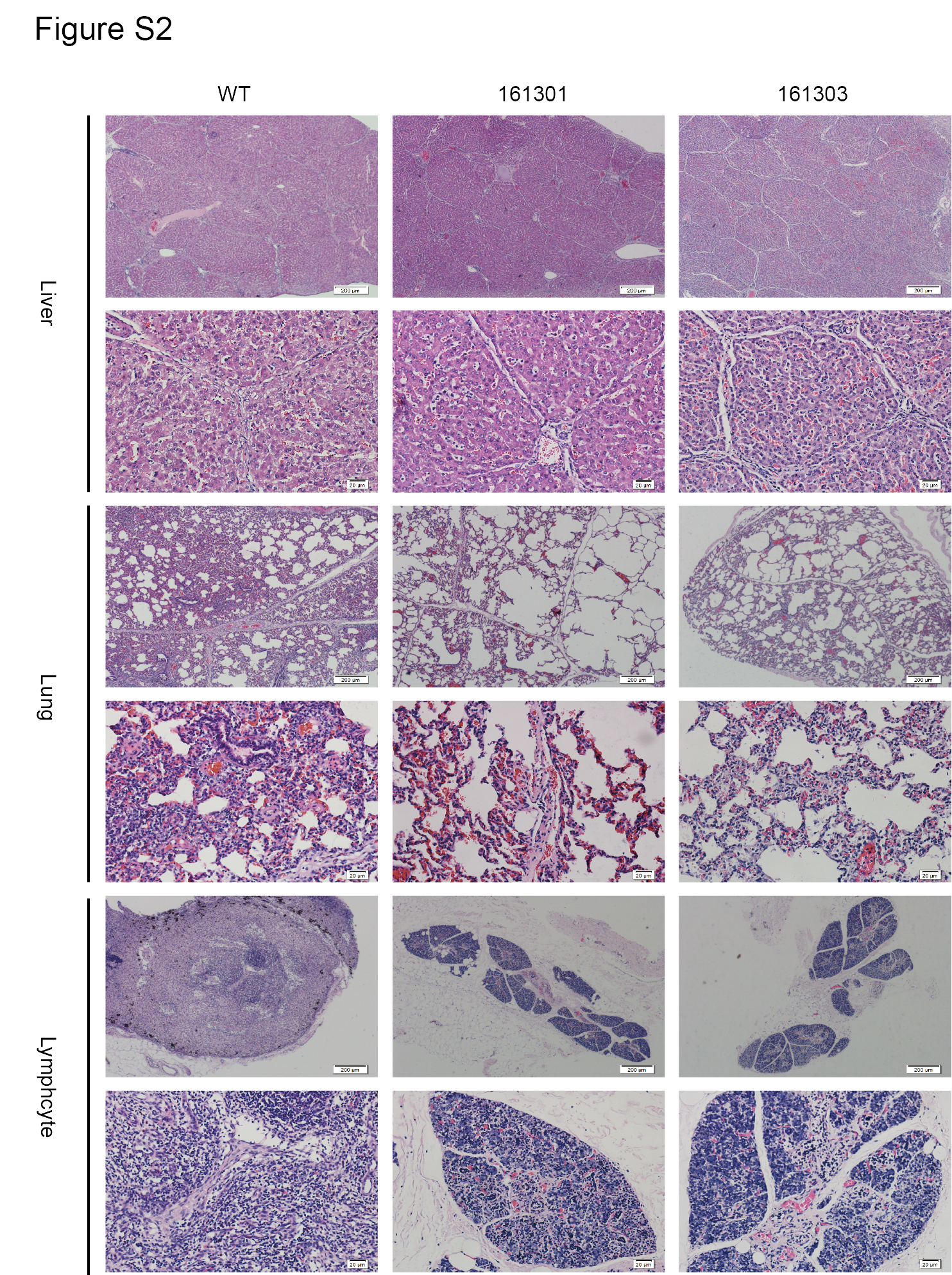

Supplement: Supplementary file 2 — Figure S2 [file CPR-53-e12863-s002.tif]
